# A Starting Point on Recycling Land and Sea Snail Shell Wastes to Manufacture Quicklime, Milk of Lime, and Hydrated Lime

**DOI:** 10.3390/ma17235683

**Published:** 2024-11-21

**Authors:** Eduardo Ferraz, Denise Terroso, Maria Cristina Sequeira, Maria Celeste Azevedo, João Coroado, Carlos Monteiro, Fernando Rocha, José A. F. Gamelas

**Affiliations:** 1 TECHN&ART, Polytechnic Institute of Tomar, Quinta do Contador, Estrada da Serra, PT-2300-313 Tomar, Portugal; jcoroado@ipt.pt; 2 GEOBIOTEC, Department of Geosciences, University of Aveiro, Campus Universitário de Santiago, PT-3810-193 Aveiro, Portugal; laraterroso@ua.pt (D.T.); csequeira@ua.pt (M.C.S.); tavares.rocha@ua.pt (F.R.); 3 Department of Chemistry, University of Aveiro, Campus Universitário de Santiago, PT-3810-193 Aveiro, Portugal; cazevedo@ua.pt; 4 CaCO_3_-Conservação do Património Artístico, Lda, Vale Cabrito-Madalena, PT-2305-441 Tomar, Portugal; geral@caco3.pt; 5 CERES, Department of Chemical Engineering, University of Coimbra, Pólo II. R. Sílvio Lima, PT-3030-790 Coimbra, Portugal

**Keywords:** gastropod shell, wet slaking, reactivity, by-product, construction material, binder, green manufacturing, circular economy

## Abstract

The valorization of gastropod shell wastes in the production of lime is the topic of this study. First, shells from land snail and sea snail were characterized for their mineralogical, chemical, and thermal properties. Then, the shells were calcined at 1000 °C, and the obtained quicklimes were characterized for their specific surface area, pore diameter, and particle morphology, followed by evaluation of their reactivity in wet slaking tests. Comparisons were made with lime from limestone. It was found that both gastropod shell wastes were composed of aragonite as the dominant crystalline phase. The quicklime from land snail belonged to the most reactive class (R5) of the wet slaking reactivity, reaching 60 °C in about 5 min, whereas the quicklime from the sea snail belonged to the R4 class, reaching 60 °C in about 14 min. However, both were much less reactive than the lime from limestone (60 °C in 25 s). The lower reactivity of quicklime from sea snail shells compared to quicklime from land snail shells could be related to its higher sulfur content (as contaminant), lower pore diameter, and the presence of particles with rounder surfaces. The reference quicklime from limestone was more reactive, mainly due to the much higher specific surface area and lower particle size. It was concluded that the gastropod shell wastes can be used in lime manufacturing.

## 1. Introduction

Land snails, freshwater snails, and seawater snails are common names for very slow-moving terrestrial or aquatic animals (*Mollusca* phylum and *Gastropoda* class) with an external spiral shell. This hard shell is a biogenic calcium carbonate exoskeleton, which protects the animal from predators, mechanical damage, and dehydration, while also enclosing and supporting the soft parts of the body and serving as a site for calcium storage.

Land snail shells were used for the removal of cadmium from aqueous solutions [1]. Sea snail shells were studied as a coagulant aid in alum precipitation of aniline blue [2] and malachite green [3] and as polyester reinforcement for coating of steel pipeline [4]. Calcined seasnail shells were proposed as a transesterification catalyst for biodiesel production [5] and for the removal of phosphate from aqueous solutions [6].

Since these gastropod shells are fundamentally composed of calcium carbonate, they have the potential to be used in the manufacturing of calcitic building lime (quicklime, milk of lime and hydrated lime). Previous research was focused on the evaluation of bivalve shells [7], eggshell [8], and cuttlebone [9] for the production of air lime. The main goal of this study concerned the evaluation of the relevant differences of the wet slaking reactivity of gastropod shell wastes compared with the previously studied shell wastes from other sources. The relationship between the mineralogical, chemical, and thermal properties of the gastropod shell wastes and the respective calcined and hydrated materials was studied. A final goal was related to the production of lime materials to be used without any constraint in air lime mortars.

Recycling these wastes in the industrial lime manufacturing process has several benefits and enables some opportunities, such as the absence of costs related to the disposal tariff in landfills or to waste incineration, the optimization of limestone exploitation, the reduction of environmental impacts during the exploitation process, reductions in the energy consumed and time spent in the comminution stage of the shell wastes (compared to limestone), valorization of the calcined wastes in the construction industry, the production of an ecological binder material (lime), and attractive perspectives regarding a circular economy and cleaner manufacturing purposes.

## 2. Materials and Methods

In the present work, gastropod shell wastes from land snail, namely, Mediterranean snail (*Theba pisana*, Müller, 1774), and sea snail, namely, common whelk (*Buccinum undatum*, Linnaeus, 1758), were used.

A commercial limestone obtained from VAC Minerais, S.A. (Rio Maior, Portugal) was used as the raw material to produce commercial lime and as the reference in this study to compare the characterization data and behavior of the shell wastes. Data related to this commercial raw limestone and the laboratorial limes (quicklime, milk of lime, and powder hydrated lime) obtained from it were previously published [8].

**Figure 1:**
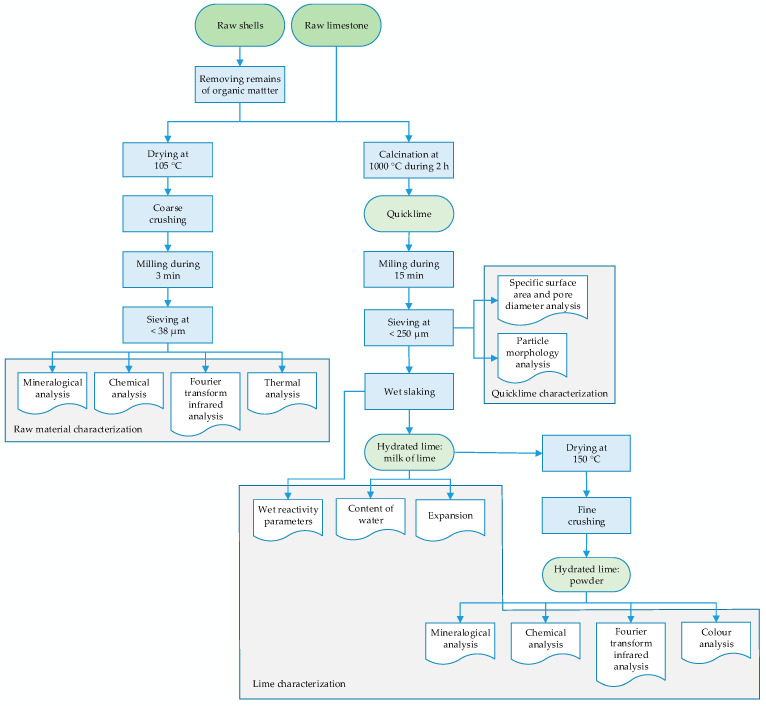
Laboratory stages and characterization of the raw materials, quicklimes, and hydrated limes (adapted from [7]).

The laboratory stages for the characterization of the gastropod shell wastes and raw limestone, as well as the obtained lime products, are presented in Figure 1.

To dissolve the remains of organic matter located inside the raw shells, these shells were immersed for 3 days in a 1:1 (*v*/*v*) NH_3_ 25%:H_2_O_2_ 35% solution that was manually stirred until the end of effervescence and further washed with demineralized water.

The raw shell wastes and limestone were dried in a Memmert UF110 oven (Memmert GmbH & Co. KG, Schwabach, Germany) at 105 ± 5 °C, coarsely crushed in a porcelain mortar, milled in a Retsch RMO 100 tungsten grinder (Retsch GmbH, Haan, Germany) for 3 min, and sieved at 38 µm to obtain powder samples.

The raw material powder samples were characterized by mineralogical, chemical, spectroscopic, and thermal analyses.

The mineralogical analysis, carried out on non-oriented (random) powder samples, was performed on a Philips X’Pert PRO MPD diffractometer (Philips N.V., Amsterdam, The Netherlands) using the X-ray diffraction (XRD) technique, operating with CuKα radiation at 50 kV and 30 mA. The intensity was determined by counting with a scan rate 0.02 °θ/s in the range 4°–60° 2θ. The identification of the crystalline phases was made by comparison with the powder diffraction files (PDF) from the International Centre for Diffraction Data (Newtown Square, PA, USA).

The chemical analysis of the major and minor elements was carried out by wavelength dispersive X-ray fluorescence (XRF) spectrometry using a PANalytical PW 4400/40 Axios equipment (PANalytical N.V., Almelo, The Netherlands) with CrKα radiation. A disc (~4 cm diameter and ~0.5 cm height), shaped in a Graseby 15T manual hydraulic press (Specac Ltd., Orpington, UK) and pressed at ~115 MPa, was previously prepared with ca. 10 g of powder and 4–5 drops of polyvinyl alcohol. The loss on ignition (LOI) was determined by gravimetric analysis, after calcinating the sample in a Carbolite CSF 1200 electric furnace (Carbolite Ltd., Derbyshire, UK) at 1000 °C for 3 h in oxidizing atmosphere.

Fourier transform infrared (FTIR) analysis was accomplished in a Bruker Alpha spectrometer (Bruker Optik GmbH & Co. KG, Ettlingen, Germany) using single attenuated total reflection (ATR) mode, 4 cm^−1^ resolution, 128 scans, and a 400–4000 cm^−1^ spectral range.

Thermogravimetric (TG) analysis and differential scanning calorimetry (DSC) were performed simultaneously on a Netzsch Jupiter STA 449C apparatus (Netzsch GmbH, Selb, Germany) under an oxidizing atmosphere between 20 and 1000 °C at a heating rate of 10 °C/min.

For the lime characterization, the raw shell wastes and limestone were calcined in a Nabertherm N 100/H laboratory kiln (Nabertherm GmbH, Lilienthal, Germany) at 1000 °C, with a 2 h soaking period in an air atmosphere and a heating rate of 3 °C/min.

The quicklime was milled in an Eti A0672 porcelain roller ball mill (Proeti S.A., Algete, Spain) for 15 min and sieved under 250 µm. The product was submitted to specific surface area, pore diameter (4 V/A), and particle morphology analyses.

The specific surface area and average pore diameter (4 V/A) of the quicklime samples were determined by N_2_ adsorption at 77 K in a Micromeritics ASAP 2000 instrument (Micromeritics Instrument Corporation, Norcross, GA, USA) using the BET theory. Samples were previously treated in a vacuum at room temperature before analysis.

The morphology of the quicklime particles was evaluated using field emission scanning electron microscopy (FESEM) in a Carl Zeiss Merlin microscope (Carl Zeiss AG, Oberkochen, Germany) in secondary electron mode. Samples were previously sputter-coated with gold using an Edwards EXC 120 turbo vacuum pump controller (Edwards Ltd., Burgess Hill, UK) coupled with a Huttinger PFG 1500 DC generator (Trumpf Hüttinger GmbH & Co. KG, Freiburg, Germany) for direct current sputtering.

The quicklime powders were submitted to the wet slaking reactivity test, in accordance with [10]. The reactivity test was carried out using a Heidolph RZR 2102 stirrer (Heidolph Instruments GmbH & Co. KG, Schwabach, Germany) at 300 ± 10 rpm and an Omega HH376 Data Logger (Omega Technology Inc., New Taipei City, Taiwan) with a resistance temperature detector (RTD) probe thermometer to record the temperature of the lime suspension every 2 s during the 35 min duration of the running test. The wet slaking curve was calculated with the average data from three replicates, considering for each sampling time a coefficient of variation in the measured temperatures below 10%.

The milks of lime, obtained from the wet slaking tests, were characterized concerning the content of water and expansion behavior, according to [10].

The content of water was determined by gravimetric difference after drying ~20 mL of milk of lime at 105 ± 5 °C in a Memmert UF110 oven (Memmert GmbH & Co. KG, Schwabach, Germany).

The expansion was evaluated by visual inspection of expansion cracks on two cakes that were ~60 mm in diameter and ~10 mm in thickness. The cakes were obtained after drying the milk of lime at 105 ± 5 °C in a Memmert UF110 oven (Memmert GmbH & Co. KG, Schwabach, Germany) for 4 h. The milk of lime was previously poured into a cylinder mold over an absorbent plate of hydrated calcium silicate.

The hydrated lime powder samples, after drying at 150 ± 5 °C in a Memmert UF110 oven (Memmert GmbH & Co. KG, Schwabach, Germany) and manual crushing in a porcelain mortar, were characterized by mineralogical analysis with XRD, chemical analysis with XRF spectrometry, and spectroscopic analysis with FTIR-ATR spectroscopy, as described above. In addition, color analysis was also performed.

The color was evaluated using a portable Datacolor Check 3 spectrophotometer (Datacolor AG, Lucerne, Switzerland) under the following conditions: d/8° measuring geometry, 2 nm wavelength resolution, 10 nm effective bandwidth, and spectral range from 360 to 700 nm (UV included). D65 illuminant, 10° observer, no gloss compensation, specular component exclusion, and small area view aperture (10.0 mm) were selected. The color results expressed in CIE L*a*b* color space were calculated with the data obtained from four replicates.

## 3. Results and Discussion

### 3.1. Characterization of the Raw Limestone and Raw Gastropod Shell Wastes

The raw limestone and raw shell wastes were evaluated according to mineralogical, chemical, FTIR, and thermal characterization.

**Figure 2:**
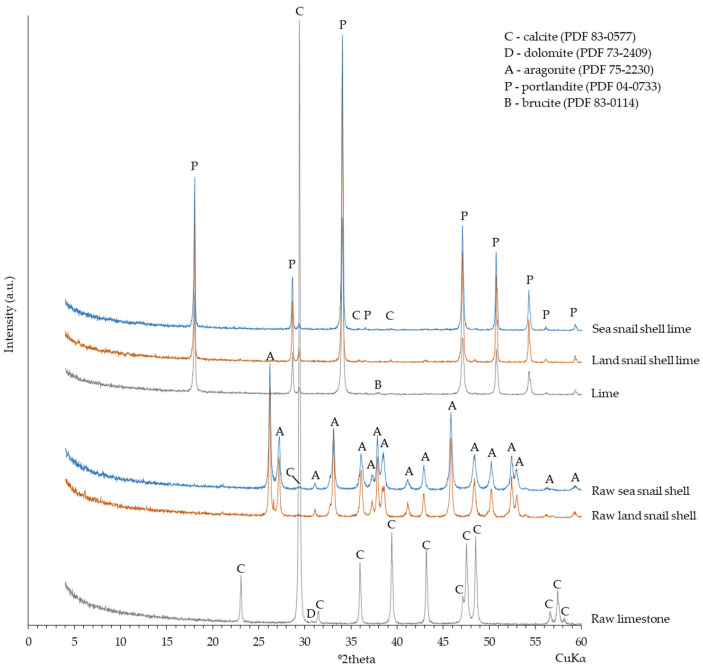
Diffractograms of the raw limestone, raw gastropod shell wastes, and powder hydrated limes.

Figure 2 shows the XRD patterns, and the identified crystalline phases of the commercial (raw) limestone and the (raw) gastropod shell wastes. Raw limestone is predominantly composed of calcite (CaCO_3_), with a vestigial content of dolomite (CaMg)(CO_3_)_2_ [7]. In both gastropod shells, the dominant presence of aragonite (CaCO_3_) with traces of calcite was observed. Aragonite is a polymorph of calcium carbonate and naturally occurs (solely or joint with calcite) in subterranean [11], freshwater [12], and seawater [13] gastropod shells.

The major chemical element determined in the raw materials using XRF (Table 1) was Ca (53–55%), as expected, supporting the presence of calcium carbonate identified by XRD as calcite and aragonite crystalline phases. Additionally, the raw limestone showed 0.4% of Mg, which is correlated with the vestigial dolomite identified by XRD. The S detected in the raw sea snail shell (0.3%) is probably related to organic matter or associated with inorganic contaminants, since it was not found any sulfur-containing mineral phase using XRD. The same interpretation could be made for Na (0.2% and 0.6%, for land snail and sea snail shells, respectively). All the raw samples presented LOI values from 44 to 46%, predominantly related with the CO_2_ release from calcite, dolomite, and aragonite identified by XRD. For the gastropod shells, the release of some remaining organic matter that was not completely removed in the preparation of the samples also contributes to the LOI values.

As for the minor chemical elements (Table 2), for land snail and sea snail shells, the determined Cl content (178 and 190 ppm, respectively) is related to the seawater salinity. However, Sr (400 and 1398 ppm, respectively) displayed relevant values. A propensity for Sr assimilation on bivalve shells was also observed in [7,14,15].

**Figure 3:**
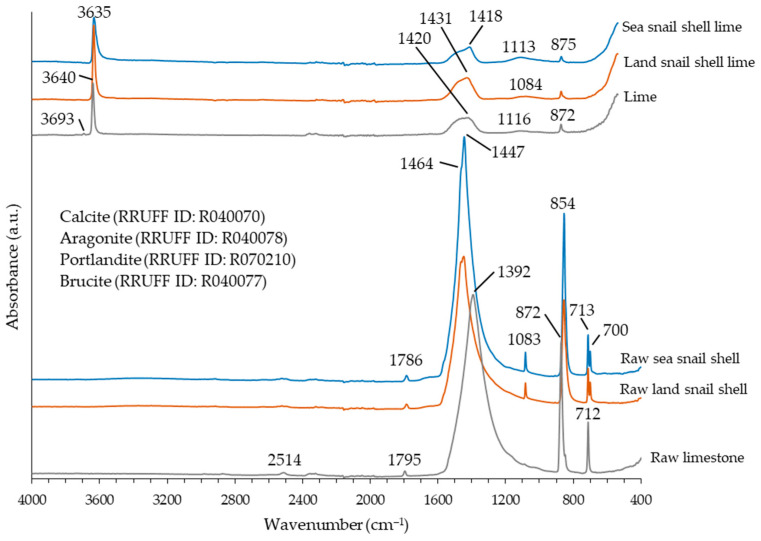
FTIR spectra of the raw limestone, raw gastropod shell wastes, and powder hydrated limes.

Figure 3 depicts the FTIR spectra of the studied materials. As previously reported in [7], the raw limestone shows the high-intensity bands of calcite at 1392 cm^−1^ (ν3), 872 cm^−1^ (ν2), and 712 cm^−1^ (ν4), corresponding to vibration modes of the C–O bonds in carbonate group, and the low-intensity bands at 2514 cm^−1^ (2ν2 + ν4) and 1795 cm^−1^ (ν1 + ν4), which are harmonic vibrations of the fundamental modes. The spectra of the two gastropod shells clearly showed the fundamental bands attributed to aragonite, which are a doublet at 1464 (ν3a) and 1447 cm^−1^ (ν3b), bands at 1083 cm^−1^ (ν1) and 854 cm^−1^ (ν2), and another doublet at 713 cm^−1^ (ν4a) and 700 cm^−1^ (ν4b). One harmonic vibration at 1786 cm^−1^ (ν1 + ν4) related to aragonite was also observed. The obtained results are in concordance with those reported in [16,17]. The results obtained by FTIR analysis corroborate the XRD results regarding the major mineral phases present in the raw materials.

**Figure 4:**
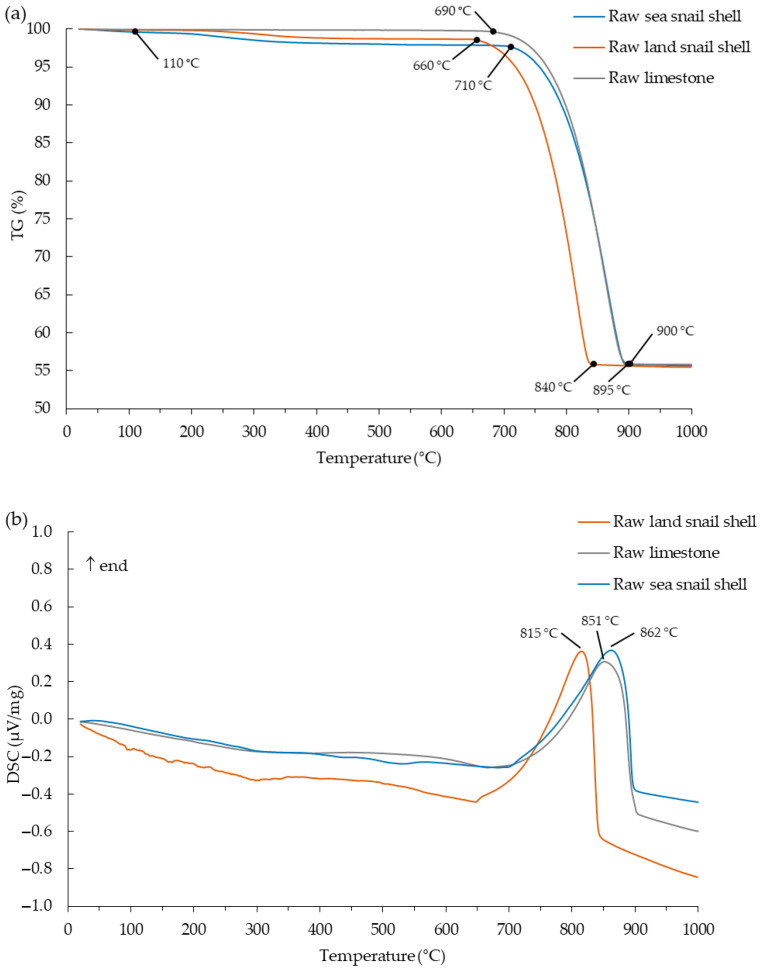
Thermal analysis patterns of the raw limestone and raw gastropod shell wastes: (**a**) Thermogravimetry (TG); (**b**) Differential scanning calorimetry (DSC).

The raw gastropod shell samples showed a similar thermogravimetric behavior (Figure 4a), with slight differences in the onset of the thermal degradation of calcium carbonate, and the respective weight loss could be divided in three steps. The first up to ~110 °C is attributed to the release of adsorbed moisture (0.1% for land snail shell and 0.4% for sea snail shell). The second step developed between ~110 and ~400 °C is ascribed to the release of the residual organic matter present in the seashells, with weight losses of 1.1% (land snail shell) and 1.5% (sea snail shell). The most relevant weight loss, due to the thermal degradation of calcium carbonate to calcium oxide (release of CO_2_), was 42.7% (~660 to ~840 °C) for land snail shell and 41.8% (~710 to ~895 °C) for sea snail shell. For this last step, the DSC pattern showed an intense endothermic peak (Figure 4b). This step occurred at different temperatures for the two gastropod shells, starting at ~660 °C for the land snail shell and at ~710 °C for the sea snail shell, showing the higher thermal stability of the sea snail shell. This higher thermal stability is comparable with the thermal stability obtained for edible cockle shell [8], which presented an onset temperature of calcium carbonate degradation of ca. 700 °C for a similar mineralogical and chemical composition. The values for the total weight loss (44.5% for land snail shell, 44.3% for sea snail shell, and 44.2% for raw limestone) were close to the LOI values obtained.

### 3.2. Characterization of the Quicklimes

The obtained quicklimes were evaluated for their specific surface area, pore diameter, and particle morphology. The BET specific surface areas were 5.2, 0.8, and 1.0 m^2^/g, and the BET average pore diameter (4 V/A) values were 14.1, 12.6, and 10.7 nm for the quicklimes from limestone, land snail shell, and sea snail shell, respectively. As expected, the calcium oxide from limestone showed the highest value for the specific surface area, while the calcium oxides from the two gastropod shells displayed comparable values with a slightly higher value for the sea snail shell. Comparing the average pore diameter, the calcium oxide from land snail shell showed a slightly higher value than that of sea snail shell.

**Figure 5:**
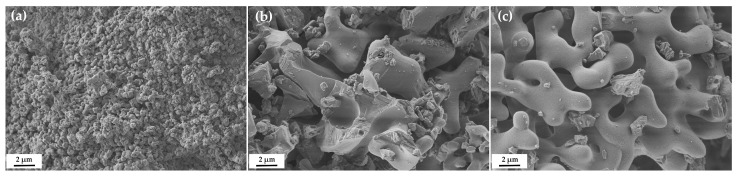
FESEM microphotographs (5000× magnification) of the quicklimes from the following: (**a**) Limestone; (**b**) Land snail shell waste; and (**c**) Sea snail shell waste.

The FESEM images portray differences in the morphology and size of the CaO particles (Figure 5). It can be observed that particles of CaO from limestone (Figure 5a) are smaller, when compared with the other two CaO materials (Figure 5b,c). In general, for all samples, the shape of the particles is rounded with smooth and undulated surfaces. In detail, some CaO particles from land snail shell presented “broken edges” with uneven and angular surfaces (Figure 5b). All the quicklimes exhibited “botryoidal-voids-like agglomeration”, promoted by an interconnection of individual “botryoid” units into a porous framework, as well denoted in Figure 5b,c.

### 3.3. Characterization of the Milks of Lime

The quicklimes were evaluated for their wet reactivity, and the obtained milks of lime were characterized for their content of water and expansion behavior.

**Figure 6:**
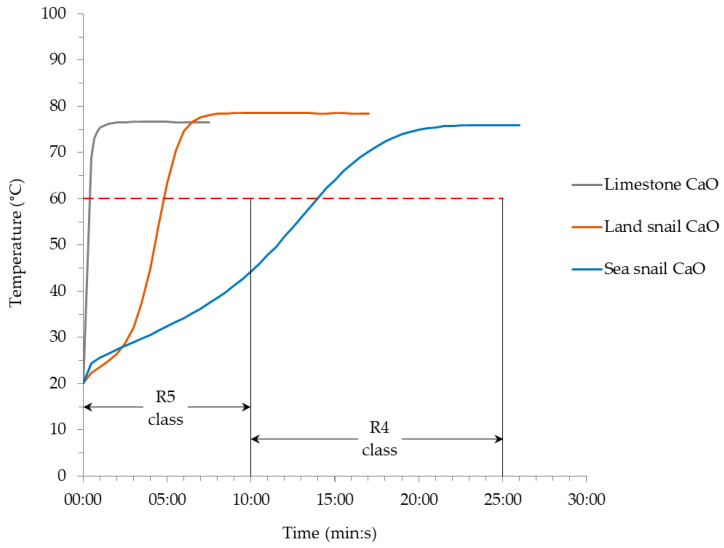
Wet slaking curves of the quicklimes from limestone and gastropod shell wastes. Horizontal red dotted line: temperature threshold of t60; vertical black solid lines: time limits of the reactivity classes.

The wet reactivity parameters and content of water are denoted in Table 3, and the wet slaking curves (the cooling of the suspensions is not drawn) are shown in Figure 6.

The quicklime from limestone has the fastest slaking time (60 °C in 25 s), classifying this material as the most reactive in the R5 class (reach 60 °C in less than 10 min) [7]. The quicklime from land snail shell is still in the R5 class (60 °C in 04:49 min:s), but the quicklime from sea snail shell was less reactive (60 °C in 13:57 min:s), which is consistent with its position in the R4 class (reach 60 °C between 10 and 25 min).

The quicklime from sea snail shell produced spheroidal lumps during the wet slaking, a fact that can have a negative influence on the reactivity of this material. The same issue was observed for calcined scallop, mussel, and oyster shells, in which the lumps were composed of portlandite [Ca(OH)_2_] and calcite [7]. The S content (0.3%) present in the raw sea snail shell (Table 1) may also have a detrimental effect on the wet slaking reactivity, as reported in [18]. The same trend was noted in [7] for calcined shells from clam, oyster, and scallop.

As reported in [19] for calcined limestone and observed in [7] for calcined bivalve shells, the distinction between calcite and aragonite in the carbonate raw materials is not a key parameter for relevant wet reactivity differences.

The slaking behavior of the quicklimes from the studied shells was entirely different from that of the quicklime from limestone. The latter exhibited a curve with a “straight” pattern in contrast to the “S” pattern for the shells: land snail with a “narrow S” and sea snail with a “broad S” (Figure 6). As previously reported in [8], the hydration of the quicklime from limestone is developed in one step at a very high rate (97.7 °C/min, 00:00 to 00:30 min:s). In this case, the wet slaking curve does not illustrate the individualization of the induction (or retardant) periods identified in [20,21,22,23,24,25,26] and by others. On the other hand, the hydration of the quicklimes from gastropod shells presented three steps, with the respective hydration rates that are correlated with three induction periods. The first step has a hydration rate of 4.6 °C/min (00:00 to 00:30 min:s) for land snail shell and of 8.3 °C/min (00:00 to 00:30 min:s) for sea snail shell. This step could be associated with the first induction period that is connected with the dissolution of calcium oxide due to the formation of thin layers of portlandite on the surface of the calcium oxide particles. The second step comprised the lowest hydration rates, of 3.0 °C/min (00:30 to 02:30 min:s) for land snail and 2.0 °C/min (00:30 to 10:00 min:s) for sea snail, and could be associated with the second induction period, which is related with the precipitation of portlandite from the supersaturated liquid phase. Finally, the last step, occurring at the later stage of the hydration process, represents the third induction period. This period is a “mix” of the behaviors of the first and second induction periods developed at the same time. In this step, the hydration rates presented values of 14.3 °C/min (02:30 to 06:00 min:s) for land snail and 3.9 °C/min (10:00 to 17:00 min:s) for sea snail. For this last step, and in the case of the land snail shell a much higher hydration rate (14.3 °C/min) than that of the first step was achieved. This means that in this third induction period, the behavior of the first induction period (in general, the most reactive with the highest slaking rate) “overlaps” over the behavior of the second one (the less reactive with the lowest slaking rate). Consequently, the dissolution of CaO “overlaps” over the precipitation of Ca(OH)_2_ from the supersaturated liquid. Additionally, it should be highlighted the Tmaximum (Table 3) achieved by the quicklime from land snail shell (85.8 °C), which represents an increase of 4.3% compared to the quicklime from limestone (82.3 °C). It was the highest Tmaximum value obtained considering all the studied shell wastes [7,8,9]. This detail could be related to the mentioned hydration rate achieved in the third step, acting like a “heat booster” at the end of the slaking process. The “broad S” pattern obtained for the sea snail shell followed the typical “S” pattern observed for calcined bivalve seashells [7].

During the wet slaking test, no significant variations in the thickening of the quicklime suspensions were detected.

The specific surface area [19], particle size [20,26], and pore diameter of the quicklimes are key parameters affecting the wet slaking reactivity. In this study, the highest reactivity for the calcium oxide from limestone must be related to the highest specific surface area and average pore diameter (5.2 m^2^/g and 14.1 nm, respectively). Additionally, the calcium oxide from limestone showed the lowest particle size, as assessed using FESEM, while the calcium oxides from the gastropod shells presented larger and similar particle sizes (to each other). Comparing the calcium oxides from the two shells, the difference in their reactivity could be related to the slightly higher average pore diameter (4 V/A) for land snail shell (12.6 nm), when compared to sea snail shell (10.7 nm). Finally, the irregular surfaces of some particles of quicklime from land snail shell could also promote a higher reactivity in the wet slaking, when balanced with the quicklime from the other shell.

The suspensions obtained after the wet slaking test presented a content of water, also called free water (Table 3), of 73.0% for land snail shell, 73.4% for sea snail shell, and 72.0% for limestone. These values classify them as milks of lime, according to [27]. The milk of lime is a consequence of using 150 g CaO:600 g H_2_O for carrying out the wet reactivity test, as established in [10].

**Figure 7:**
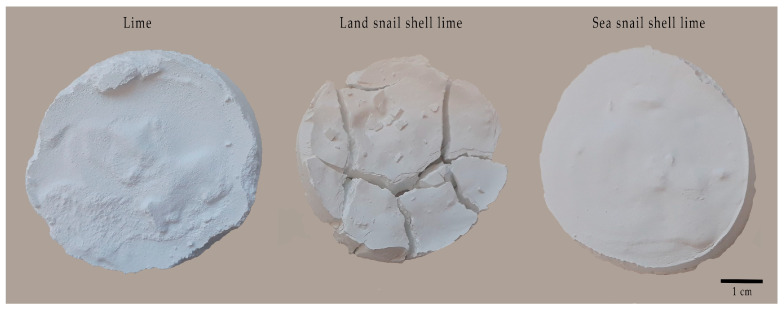
Expansion behavior of the milks of lime from limestone and gastropod shell wastes.

The milks of lime did not present expansion behavior (Figure 7 and Table 3), although the lime from land snail shell exhibits shrinkage cracks, which does not imply a disadvantage.

### 3.4. Characterization of the Powder Hydrated Limes

The quality of the obtained hydrated limes in dry powder form was evaluated according to mineralogical, chemical, FTIR, and color characterization.

Portlandite was the main mineral phase detected in all limes, together with some vestigial content of calcite (Figure 2), mainly due to the limes’ carbonation from wet to dry form.

In the lime from limestone, traces of brucite [Mg(OH)_2_] were also detected (Figure 2), which were formed by the hydration of MgO that originated from the calcination of dolomite present in the raw limestone [7].

The chemical analysis of the powder hydrated limes (Table 1) showed a large Ca content (76% for lime from land snail shell and 74% for lime from sea snail shell) related to the presence of portlandite and calcite, as identified by XRD (Figure 2). The Mg content (1.3%) present in the lime from limestone was related to the brucite presence identified by XRD (Figure 2). Additionally, the hydrated lime from sea snail shell showed 0.4% in S content, whereas the respective lime from land snail shell showed less than 0.1% (Table 1). Previously, a reasonable positive linear correlation between this chemical element and the increase in the t60 parameter was found [7]. Since sulfur is a penalizing element in the wet slaking process, the larger content of sulfur in the lime from sea snail shell could also have contributed to the lower reactivity of this lime in the wet slaking (higher t60). Finally, the LOI values for the hydrated limes from the gastropod shells were higher than that of the lime from limestone (23% for land snail, 24% for sea snail, and 16% for the lime from limestone).

Similarly to the raw shells, Cl (70 ppm for land snail shell and 50 ppm for sea snail shell) and Sr (480 ppm for land snail shell and 1970 ppm for sea snail shell) were found as minor chemical elements in the limes (Table 2).

The FTIR spectra (Figure 3) showed a strong band at 3635 cm^−1^ in the limes from the gastropod shells (3640 cm ^−1^ in the lime from limestone) related with the OH stretching in portlandite [17,28]. Medium/weak intensity bands from calcite formed from the fresh carbonation of portlandite were also observed at ~1418–1431 cm^−1^ and 875 cm^−1^. A broad band with weak intensity at ~1084–1113 cm^−1^ was also present in the spectra of the hydrated lime samples that could be related to the presence of sulfate ions [29], namely for the lime from sea snail shell (0.4% in S content), and agrees well with the reported spectra for other limes obtained from bivalve shells [7].

Table 4 presents the color parameters of the powder hydrated limes. The difference in the chromatic L*a*b* parameters between the lime from limestone and the limes from gastropod shells is small. Notwithstanding, when compared with the reference lime, the lime from sea snail shell is slightly lighter (ΔL* = 0.2), and the lime from land snail shell is slightly darker (ΔL* = −0.2). A slight shift toward green (Δa* = −0.1) was observed for the lime from land snail shell. Both shell limes showed a small shift to the blue (Δb* = −0.6 and −0.7 for land snail and sea snail, respectively). For small variations, the total color difference according to CIE deltaE2000 (ΔE00) with k_L_ = k_C_ = k_H_ = 1 [30] is more accurate and more adapted to the perception of the human eye [31]. The total color difference of the gastropod shell limes (0.6 and 0.7 for land snail and sea snail, respectively) was insignificant and imperceptible to human eye.

## 4. Economic, Engineering, Industrial, Environmental, and Technological Implications

A modern lime factory must be supplied with ~1650 t/day of limestone to produce ~920 t/day of quicklime. In Portugal, although land snail shell wastes are generated by the food industry (frozen boiled crumb), their annual production is not statistically quantified. As for the sea snail, there is no Portuguese production of related food products. Notwithstanding, assuming a maximum average production of 0.1 t/day of shell wastes, this value would represent around 0.006 wt.% of the limestone that could be replaced in the lime production process.

The elimination of these shell wastes may cost ~150 EUR/t (to treat, store, load, transport and deposit in landfill or incinerate) for a maximum delivery distance of 150 km. This type of elimination has the consequent ecological and economic constraints.

A critical factor is related to industrial calcination, since the modern lime factories have installed parallel flow kilns, that currently are not adapted to being fed with small grain size (<30 mm). Even more relevant, the heat flow and pressure inside the kiln chamber will be disturbed due to the turbulence promoted by the presence of small grains.

Another disadvantage, during the industrial calcination stage, is related to the decomposition of the organic matter presented in the shell wastes. This fact is expected to increase the emissions of volatile organic compounds, which in the lime factory must comply with the respective legal limits.

Concerning hygiene and safety conditions, the lime factory must be legally authorized for the management of these types of waste and needs to make the necessary lay-out adaptations to receive, store, and manipulate the wastes.

Recycling these shell wastes in the production of a binder material contributes, at the same time, to the requirements for sustainable construction. The recycling increases private and public companies’ awareness for sustainable waste elimination and could generate an opportunity within the context of a circular economy and greener manufacturing.

Finally, as previously indicated for calcined limestones and dolomites [18], fast slaking does not necessarily mean the best binder performance of the calcined material when used in air lime mortars because other relevant variables will affect the hardening of this system. Future studies should be carried out in the laboratory and on-site for mortars formulated with the limes from shell wastes to evaluate their performance.

## 5. Conclusions

The present study aimed the valorization of land snail and sea snail shell wastes by exploring their potential to produce calcitic lime for construction. The mineralogical, chemical, FTIR, and thermal characterization of the gastropod shell wastes discriminated aragonite, as the main crystalline phase present.

The wet slaking tests showed that the quicklime obtained from the land snail shell waste belongs to the most reactive class (R5: reaching 60 °C in 04:49 min:s), which is the same class of the quicklime from limestone (R5: reaching 60 °C in 00:30 min:s), but the quicklime from sea snail shell belonged to a less reactive class (R4: reaching 60 °C in 13:57 min:s).

The wet slaking curves for the gastropod shell quicklimes followed a typical ‘‘S’’ pattern with a “broad S” for sea snail shell and a “straight S” for land snail shell. The first one is closer to the common behavior observed for seashells, comprising a first stage (00:30 min:s) with a higher hydration rate (8.3 °C/min) and a long third stage (07:00 min:s) with an intermediate hydration rate (3.9 °C/min). On the other hand, a less usual behavior was observed for the lime from land snail shell, comprising a first stage (00:30 min:s) with an intermediate hydration rate (4.6 °C/min) and a long third stage (03:30 min:s) with the highest hydration rate (14.3 °C/min).

This distinct slaking behavior exhibited by the two gastropod shell limes could be related to differences in the morphology and BET average pore diameter (4 V/A) of the calcium oxide particles. The slightly higher average pore diameter for quicklime from land snail shell compared to that of quicklime from sea snail shell and the irregular surfaces of some particles of quicklime from land snail shell have promoted a higher reactivity in the wet slaking for the latter. The presence of a significant amount of sulfur in the quicklime from sea snail shell may also have inhibited the reactivity of this quicklime compared to the quicklime from land snail shell, since sulfur is a penalizing element in the process. However, the reactivity of both limes from gastropod shells was lower than that of lime from limestone. The highest BET specific surface area, highest BET average pore diameter, and the smallest particle size for the quicklime from limestone translated into a remarkably higher reactivity of this reference lime in the wet slaking process.

Finally, the powder hydrated limes from gastropod shells presented mineralogical, chemical, and color properties similar to the lime produced from raw limestone.

The working hypothesis concerning the existence of differences in the wet slaking behavior of the gastropod shell wastes compared with other shell wastes was confirmed. It can be concluded that the studied gastropod shell wastes can be used, without any constraints, in lime manufacturing.

## Figures and Tables

**Table 1:** XRF data (major elements) of the raw limestone, raw gastropod shell wastes, and powder hydrated limes.

	CaO (%)	MgO (%)	SO_3_ (%)	SiO_2_ (%)	Al_2_O_3_ (%)	Fe_2_O_3_ (%)	Na_2_O (%)	K_2_O (%)	P_2_O_5_ (%)	LOI (%) ^a^
Raw material										
Raw limestone ^b^	55.25	0.38	0.06	0.20	0.12	0.06	0.04	0.01	0.01	43.88
Raw land snail shell	55.23	0.03	0.02	0.05	0.02	0.01	0.23	0.09	0.01	44.31
Raw sea snail shell	53.04	0.05	0.27	0.15	0.07	0.03	0.59	0.02	0.06	45.72
Powder hydrated lime										
Lime ^b^	81.73	1.25	0.16	0.22	0.11	0.17	0.09	0.04	0.01	16.24
Land snail shell lime	76.10	0.07	0.07	0.21	0.07	0.01	0.20	0.01	0.07	23.20
Sea snail shell lime	74.16	0.13	0.42	0.54	0.13	0.04	0.20	0.01	0.06	24.32

^a^ LOI: loss on ignition; ^b^ Published by [8].

**Table 2:** XRF data (minor elements) of the raw limestone, raw gastropod shell wastes, and powder hydrated limes.

Element	Raw Material			Powder Hydrated Lime		
(ppm)	Limestone ^a^	Land Snail Shell	Sea Snail Shell	Lime ^a^	Land Snail Shell Lime	Sea Snail Shell Lime
Cl	nd	178.4	190.4	185.4	70.0	50.0
Sc	9.4	16.1	12.5	8.4	7.7	7.7
Cr	6.6	6.7	10.2	8.2	11.4	10.7
Mn	27.0	17.5	6.1	60.6	28.0	9.4
Co	8.3	9.5	nd	nd	nd	nd
Ni	nd	6.9	nd	nd	nd	3.4
Cu	10.8	10.7	9.6	11.5	20.0	20.0
Zn	2.8	4.7	11.5	nd	30.0	50.0
Ga	nd	1.3	nd	nd	nd	nd
As	nd	38.9	4.4	nd	nd	nd
Se	nd	7.2	nd	nd	nd	1.8
Br	nd	6.3	43.4	0.9	nd	1.0
Rb	4.4	3.2	6.6	2.9	4.3	6.5
Sr	101.3	400.1	1347.5	160.4	480.0	1970.0
Y	1.2	nd	nd	2.7	0.9	nd
Nb	nd	1.2	nd	nd	nd	nd
Mo	2.1	2.0	1.7	2.2	2.5	2.7
Cd	nd	nd	nd	nd	8.2	nd
Sn	nd	5.1	nd	nd	nd	nd
I	nd	nd	15.6	nd	nd	nd
Cs	8.6	8.4	8.5	nd	6.7	7.3
Ba	nd	nd	nd	nd	14.9	nd
La	14.6	nd	10.9	11.3	17.6	11.4
Ce	30.8	18.9	36.6	46.0	50.0	60.0
Nd	15.5	17.9	17.9	24.6	29.0	28.0
Ta	nd	9.0	nd	nd	nd	nd
W	11.2	51.7	5.1	nd	nd	nd
Tl	nd	16.0	4.0	nd	nd	nd
Pb	8.9	18.0	3.8	3.9	40	10
Bi	nd	15.9	nd	nd	nd	nd
Th	nd	nd	7.7	nd	5.3	29.4
U	2.5	3.8	5.6	1.4	2.2	5.5
Yb, Hf, Sm, Sb, Te, Ag, Zr, Ge, V, and Ti	nd	nd	nd	nd	nd	nd

^a^ Published by [8]; nd—not detected.

**Table 3:** Wet slaking parameters of the quicklimes from limestone and gastropod shell wastes, and content of water and expansion behavior of the obtained milks of lime.

Quicklime	t60 (min:s) ^a^	Reactivity Class	T′ Maximum (°C) ^b^	T Maximum (°C) ^c^	Tu, 80% Reaction (°C) ^d^	tu, 80% Reaction (min:s) ^e^	Content of Water (%)	Expansion [Yes or No]
Limestone ^f^	00:25 ± 00:02	R5	76.6 ± 1.4	82.3 ± 1.5	65.3 ± 1.1	00:28 ± 00:02	72.0 ± 1.1	No
Land snail shell	04:49 ± 00:15		79.9 ± 2.2	85.8 ± 2.5	67.9 ± 1.9	05:18 ± 00:19	73.0 ± 0.5	
Sea snail shell	13:57 ± 01:09	R4	75.9 ± 1.9	81.5 ± 2.1	64.8 ± 1.5	15:10 ± 01:01	73.4 ± 1.0	

^a^ Time to reach 60 °C. ^b^ Maximum temperature reached during the slaking test. ^c^ Maximum temperature reached during the slaking test corrected using the following equation: T maximum = (1.1 × T′ maximum) − 2. ^d^ Temperature at which the slaking process is 80% complete, calculated using the following equation: Tu = (0.8 × T′ maximum) + (0.2 × T0), where T0 (°C) is the initial temperature of the test (~20 °C). ^e^ Time required for the slaking process to be 80% complete, obtained by the wet reactivity curve. ^f^ Published by [8].

**Table 4:** CIE L*a*b* color data for the powder hydrated limes from limestone and gastropod shell wastes, and the corresponding chromatic dif-ferences and total color difference between the gastropod shell limes and the lime from lime-stone.

Powder Hydrated Lime	L*	a*	b*	ΔL*	Δa*	Δb*	ΔE00 (1:1:1)
Lime ^a^	97.39 ± 0.62	−0.02 ± 0.01	1.21 ± 0.09	-	-	-	-
Land snail shell lime	97.17 ± 0.40	−0.14 ± 0.04	0.62 ± 0.09	−0.22	−0.12	−0.59	0.6
Sea snail shell lime	97.61 ± 0.91	−0.01 ± 0.04	0.51 ± 0.15	0.22	0.01	−0.70	0.7

^a^ Published by [8].

## Data Availability

Data are contained within the article.

## References

[1] Zhao B., Zhang J., Yan W., Kang X., Cheng C., Ouyang Y. (2016). Removal of cadmium from aqueous solution using waste shells of golden apple snail. Desalin. Water Treat..

[2] Oladoja N.A., Aliu Y.D., Ofomaja A.E. (2011). Evaluation of snail shell as a coagulant aid in the alum precipitation of aniline blue from aqueous solution. Environ. Technol..

[3] Oladoja N.A., Aliu Y.D. (2009). Snail shell as coagulant aid in the alum precipitation of malachite green from aqua system. J. Hazard. Mater..

[4] Suleiman I.Y., Aigbodion V.S., Obayi C.O., Mu’azu K. (2019). Surface characterisation, corrosion and mechanical properties of polyester-polyester/snail shell powder coatings of steel pipeline for naval applications. Int. J. Adv. Manuf. Technol..

[5] Gupta J., Agarwa M. (2019). Preparation and characterization of highly active solid base catalyst from snail shell for biodiesel production. Biofuels.

[6] Xiong J.B., Qin Y., lslam E. (2015). Adsorptive removal of phosphate from aqueous solutions by waste snail and clam shells. Environ. Eng. Manag. J..

[7] Ferraz E., Gamelas J.A.F., Coroado J., Monteiro C., Rocha F. (2019). Recycling Waste Seashells to Produce Calcitic Lime: Characterization and Wet Slaking Reactivity. Waste Biomass Valorization.

[8] Ferraz E., Gamelas J.A.F., Coroado J., Monteiro C., Rocha F. (2018). Eggshell waste to produce building lime: Calcium oxide reactivity, industrial, environmental and economic implications. Mater. Struct..

[9] Ferraz E., Gamelas J.A.F., Coroado J., Monteiro C., Rocha F. (2020). Exploring the potential of cuttlebone waste to produce building lime. Mater. Constr..

[10] (2011). Building Lime. Part 2: Test Methods.

[11] Medakovic D., Slapnik R., Grzeta B., Popovic S. (1999). The shell mineralogy of subterranean snails *Zospeum alpestre* (Freyer 1855) and *Zospeu isselianum* (Pollonera 1886) (Mollusca: Gastropoda: Carychiidae). Period. Biol..

[12] Medakovic D., Slapnik R., Popovic S., Grzeta B. (2003). Mineralogy of shells from two freshwater snails *Belgrandiella fontinalis* and *B-kuesteri*. Comp. Biochem. Physiol. A Mol. Integr. Physiol..

[13] Iglikowska A., Przytarska J., Humphreys-Williams E., Najorka J., Chełchowski M., Sowa A., Hop H., Włodarska-Kowalczuk M., Kuklińsk P. (2023). Mineralogical and chemical composition of Arctic gastropods shells. Prog. Oceanogr..

[14] Lorrain A., Gillikin D.P., Paulet Y.-M., Chauvaud L., Mercier A.L., Navez J., André L. (2005). Strong kinetic effects on Sr/Ca ratios in the calcitic bivalve *Pecten maximus*. Geology.

[15] Füllenbach C.S., Schöne B.R., Shirai K., Takahata N., Ishida A., Sano Y. (2017). Minute co-variations of Sr/Ca ratios and microstructures in the aragonitic shell of *Cerastoderma edule* (Bivalvia)—Are geochemical variations at the ultra-scale masking potential environmental signals?. Geochim. Cosmochim. Acta.

[16] Galván-Ruiz M., Hernández J., Baños L., Noriega-Montes J., Rodríguez-García M.E. (2009). Characterization of calcium carbonate, calcium oxide, and calcium hydroxide as starting point to the improvement of lime for their use in construction. J. Mater. Civ. Eng..

[17] Gunasekaran S., Anbalagan G., Pandi S. (2006). Raman and infrared spectra of carbonates of calcite structure. J. Raman Spectrosc..

[18] Boynton R.S. (1980). Chemistry and Technology of Lime and Limestone.

[19] Commandré J.-M., Salvador S., Nzihou A. (2007). Reactivity of laboratory and industrial limes. Chem. Eng. Res. Des..

[20] Adams F.W. (1927). Effect of particle size on the hydration of lime. Ind. Eng. Chem..

[21] Tadros M.E., Skalny J., Kalyoncu R.S. (1976). Kinetics of calcium hydroxide crystal growth from solution. J. Colloid. Interface Sci..

[22] Ritchie I.M., Xu B.-A. (1990). The kinetics of lime slaking. Hydrometallurgy.

[23] Giles D.E., Ritchie I.M., Xu B.-A. (1993). The kinetics of dissolution of slaked lime. Hydrometallurgy.

[24] Wolter A., Luger S., Schaefer G. (2004). The kinetics of the hydration of quicklime. ZKG Int..

[25] Rodriguez-Navarro C., Ruiz-Agudo E., Ortega-Huertas M., Hansen E. (2005). Nanostructure and irreversible colloidal behavior of Ca(OH)_2_: Implications in cultural heritage conservation. Langmuir.

[26] Kemperl J., Maček J. (2009). Precipitation of calcium carbonate from hydrated lime of variable reactivity, granulation and optical properties. Int. J. Miner. Process..

[27] (2011). Building Lime. Part 1: Definitions, Specifications and Conformity Criteria.

[28] Busing W.R., Morgan H.W. (1958). Infrared Spectrum of Ca(OH)_2_. J. Chem. Phys..

[29] Bellamy L.J. (1975). The Infrared Spectra of Complex Molecules.

[30] (2014). Colorimetry Part 6: CIEDE2000 Colour-Difference Formula.

[31] Luo M.R., Cui G., Rigg B. (2001). The Development of the CIE 2000 Colour-Difference Formula: CIEDE2000. Color Res. Appl..

